# PEG35 as a Preconditioning Agent against Hypoxia/Reoxygenation Injury

**DOI:** 10.3390/ijms23031156

**Published:** 2022-01-21

**Authors:** Rui Teixeira da Silva, Ivo F. Machado, João S. Teodoro, Arnau Panisello-Roselló, Joan Roselló-Catafau, Anabela P. Rolo, Carlos M. Palmeira

**Affiliations:** 1 Department of Life Sciences, University of Coimbra, 3000-456 Coimbra, Portugal; jteodoro@ci.uc.pt (J.S.T.); anpiro@ci.uc.pt (A.P.R.); palmeira@ci.uc.pt (C.M.P.); 2 Center for Neurosciences and Cell Biology, University of Coimbra, 3004-504 Coimbra, Portugal; imachado@cnc.uc.pt; 3 Experimental Pathology Department, Institute of Biomedical Research of Barcelona (IIBB), CSIC-IDIBAPS, 08036 Barcelona, Spain; arnau.panisello@iibb.csic.es (A.P.-R.); jrcbam@iibb.csic.es (J.R.-C.); 4 IIIUC—Institute of Interdisciplinary Research, University of Coimbra, 3030-789 Coimbra, Portugal

**Keywords:** polyethylene glycol 35, hypoxia/reoxygenation injury, mitochondria, autophagy

## Abstract

Pharmacological conditioning is a protective strategy against ischemia/reperfusion injury, which occurs during liver resection and transplantation. Polyethylene glycols have shown multiple benefits in cell and organ preservation, including antioxidant capacity, edema prevention and membrane stabilization. Recently, polyethylene glycol 35 kDa (PEG35) preconditioning resulted in decreased hepatic injury and protected the mitochondria in a rat model of cold ischemia. Thus, the study aimed to decipher the mechanisms underlying PEG35 preconditioning-induced protection against ischemia/reperfusion injury. A hypoxia/reoxygenation model using HepG2 cells was established to evaluate the effects of PEG35 preconditioning. Several parameters were assessed, including cell viability, mitochondrial membrane potential, ROS production, ATP levels, protein content and gene expression to investigate autophagy, mitochondrial biogenesis and dynamics. PEG35 preconditioning preserved the mitochondrial function by decreasing the excessive production of ROS and subsequent ATP depletion, as well as by recovering the membrane potential. Furthermore, PEG35 increased levels of autophagy-related proteins and the expression of genes involved in mitochondrial biogenesis and fusion. In conclusion, PEG35 preconditioning effectively ameliorates hepatic hypoxia/reoxygenation injury through the enhancement of autophagy and mitochondrial quality control. Therefore, PEG35 could be useful as a potential pharmacological tool for attenuating hepatic ischemia/reperfusion injury in clinical practice.

## 1. Introduction

Ischemia/reperfusion injury is a major hurdle in many clinical scenarios, including liver transplantation, hepatic resection and trauma settings [1,2]. Hepatic ischemia/reperfusion injury contributes to an increased rate of acute liver failure, graft rejection and chronic hepatic dysfunction, affecting liver surgery outcomes and patient rehabilitation [3,4]. The mechanisms of organ damage following ischemia/reperfusion have been widely studied, and involve the complex interactions of multiple pathways. Unfortunately, despite intensive research, effective therapeutic approaches for the prevention/treatment of ischemia/reperfusion injury are still clinically limited. 

Mitochondria are crucial players in every single living eukaryote. In addition to their well-established role in energetic metabolism and ATP generation, mitochondria participate in many other physiological functions, including catabolic and anabolic processes, diverse signaling pathways, calcium homeostasis and cell death mechanisms [5]. Therefore, disruptions to these processes affect normal mitochondrial function, and disruptions have been implicated in mitochondrial dysfunction. It is well established that mitochondria play a central role in ischemia/reperfusion injury [6]. The lack of oxygen observed during the ischemic period leads to a decrease in ATP levels, while the reestablishment of blood supply gives rise to ROS production, the activation of immune cells to promote inflammation and consequent cell death [5,7]. 

In physiological conditions, the maintenance of a healthy mitochondrial network is a determinant factor for cellular homeostasis and cell survival. After an ischemic event, if mitochondria remain functional enough to generate ATP, the tissue can recover and overcome the injury. Conversely, when the ischemic period is more aggressive, the subsequent restoration of blood flow and, significantly, oxygen compromises mitochondrial function, leading to an exacerbation of ROS generation. Thus, the clearance of dysfunctional mitochondria through selective degradation and their replacement by new ones via mitochondrial biogenesis, alongside changes in mitochondrial dynamics, have been suggested to be an effective quality control system to counteract hepatic ischemia/reperfusion injury and maintain mitochondrial function [8].

In the past few years, a sizeable body of literature has suggested that different drugs could play a beneficial role in avoiding ischemia/reperfusion-associated adverse effects and organ failure. Polyethylene glycols are non-toxic, neutral, water-soluble compounds approved by the Food and Drug Administration for their use in cosmetics, foods and drugs [9]. Their beneficial effects have been reported in different organs, including the liver, heart, kidney, intestine and pancreas [10,11,12,13,14]. In the liver, several studies have demonstrated the protective role that different molecular weight polyethylene glycols play during cold preservation. Polyethylene glycol 35 kDa (PEG35) has been associated with higher levels of mitochondrial aldehyde dehydrogenase 2 (ALDH2) and improved mitochondrial machinery and the subsequent diminishing of cold ischemic injury [15,16]. Furthermore, intravenous PEG35 pretreatment improved liver graft preservation and protected the mitochondria when cold ischemia was followed by warm reperfusion [17]. Besides their effects in cold storage, PEG35 has also shown hepatoprotection against warm ischemia/reperfusion injury [18]. PEG35 preconditioning efficiently reduced transaminases levels and maintained hepatocyte morphology, and also preserved mitochondrial membrane potential. 

Based on these protective features, the aim of the present study was to assess the ability of PEG35 preconditioning to protect human hepatocytes submitted to hypoxia/reoxygenation. We also explore the possible protective molecular mechanisms involved in PEG35-mediated hepatoprotection.

## 2. Results

### 2.1. PEG35 Preconditioning Increases Cell Viability

**Figure 1:**
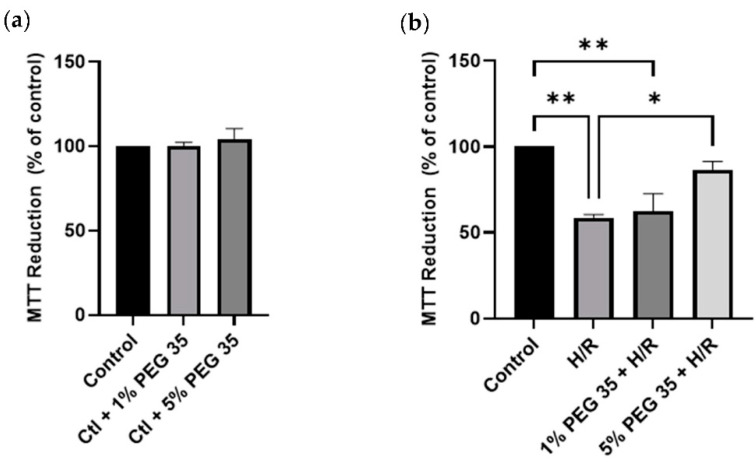
Effects of PEG35 on cell viability, in the absence (**a**) or presence (**b**) of hypoxia/reoxygenation (H/R). Cell viability rate determined via MTT assay. The values shown represent the mean ± SEM of 3 independent experiments. * *p* < 0.05; ** *p* < 0.01.

To examine the effect of PEG35 preconditioning against hypoxia/reoxygenation injury, cell viability was assessed through the reduction of a yellow tetrazolium salt to purple formazan crystals (using an MTT assay, as described in the Materials and Methods section) (Figure 1). Firstly, it was confirmed that the two different concentrations of PEG35 used throughout the study were not noxious to the cells (Figure 1a). 

The HepG2 pretreated with 5% PEG35 for 1 h and then subjected to 2 h of hypoxia followed by 2 h of reoxygenation demonstrated significantly higher cell viability compared to cells that did not receive PEG preconditioning (Figure 1b). Significantly, the protective effect of PEG35 was shown to be dose dependent, since 1% PEG35 demonstrated almost no protection against hypoxia/reoxygenation, similar to the results of the hypoxia/reoxygenation group. Strikingly, the 5% PEG35 + H/R group was able to protect HepG2 at similar levels to the control group.

### 2.2. PEG35 Administration Increases Mitochondrial ALDH2 Content and Attenuates Oxidative Injury

**Figure 2:**
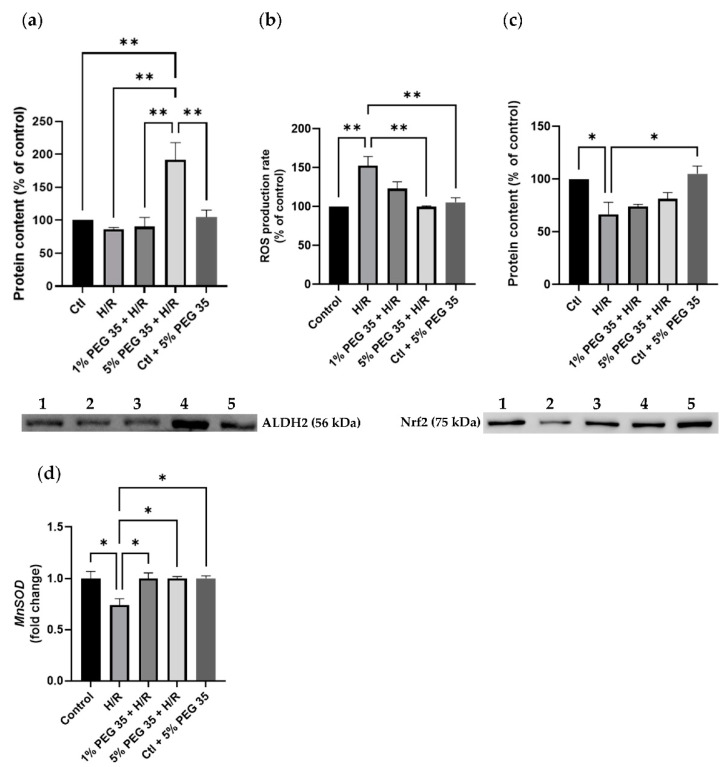
Effects of PEG 35 in mitochondrial ALDH2 levels and oxidative stress in HepG2 submitted to H/R. (**a**) Representative Western blot for ALDH2 and respective quantification for: 1-Ctl; 2-H/R; 3–1% PEG35 + H/R; 4–5% PEG35 + H/R; 5-Ctl + 5% PEG35. (**b**) ROS production rate measured fluorometrically as described in the Materials and Methods section. (**c**) Representative Western blot for Nrf2 and respective quantification for: 1-Ctl; 2-H/R; 3–1% PEG35 + H/R; 4–5% PEG35 + H/R; 5-Ctl + 5% PEG35. (**d**) Gene expression for MnSOD. The values shown represent the mean ± SEM of 3 independent experiments. * *p* < 0.05; ** *p* < 0.01.

PEG35 has been tightly linked to the mitochondrial enzyme ALDH2 when working against ischemia reperfusion injury in several studies [15,16]. Thus, we examined whether PEG35 treatment could increase ALDH2 levels. While in the control, hypoxia/reoxygenation and 1% PEG35 groups, the protein levels were similar, in the 5% PEG35 group there was a significant increase in ALDH2 content (Figure 2a). As ALDH2 is a key enzyme that functions against oxidative stress, we then investigate if its increased levels could reduce oxidative stress. As expected, compared to the control, hypoxia/reoxygenation significantly increased ROS production (Figure 2b). Moreover, in accordance with the increased ALDH2 content, the 5% PEG35 preconditioning significantly prevented the increase in ROS levels found in the hypoxia/reoxygenation group. In addition, we also evaluated the protein levels of one of the master regulators of antioxidant defense and a regulator of the expression of the mitochondrial antioxidant protein, nuclear factor-E2-related factor 2 (Nrf2) and manganese-dependent superoxide dismutase (MnSOD), respectively. The results revealed that hypoxia/reoxygenation treatment inhibited the expression of Nrf2, while the 5% PEG35 preconditioning seemed to reverse this tendency, but not in a significant way (Figure 2c). The expression of the MnSOD gene was decreased in the hypoxia/reoxygenation group, but it was recovered to control levels in the PEG35-treated groups (Figure 2d).

### 2.3. PEG35 Alleviates Mitochondrial Damage Induced by Hypoxia/Reoxygenation Injury

**Figure 3:**
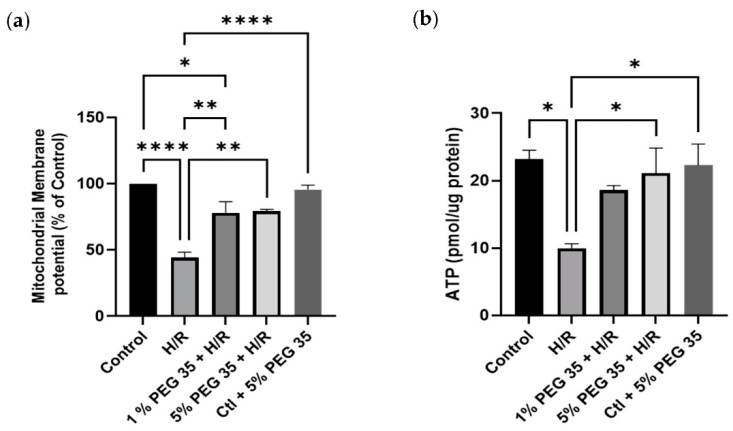
PEG35 effects on mitochondrial function. (**a**) Mitochondrial membrane potential (ΔΨ) was assessed using TMRM, as described in the Materials and Methods section. (**b**) ATP content in HepG2 cells. The values shown represent the mean ± SEM of 3 independent experiments. * *p* < 0.05; ** *p* < 0.01; **** *p* < 0.0001.

The deleterious effects associated with hepatic ischemia/reperfusion injury are well known to cause mitochondrial dysfunction. To evaluate if PEG35 preconditioning-mediated hepatoprotection during hypoxia/reoxygenation injury is due to enhanced mitochondrial dysfunction, we assessed the mitochondrial membrane potential (ΔΨ). Figure 3a clearly shows a decrease in mitochondrial ΔΨ in the cells submitted to hypoxia/reoxygenation, which was efficiently recovered in the presence of both PEG35 concentrations.

Mitochondria are the main source of ATP production, which is known to be compromised by ischemia/reperfusion injury. Thus, we next evaluated the ATP generation in all groups. As shown in Figure 3b, ATP content was decreased upon hypoxia/reoxygenation, but the administration of 5% PEG35 before hypoxia was able to significantly preserve mitochondrial ATP content.

### 2.4. PEG35 Attenuates Hypoxia/Reperfusion Injury through Enhanced Autophagy

**Figure 4:**
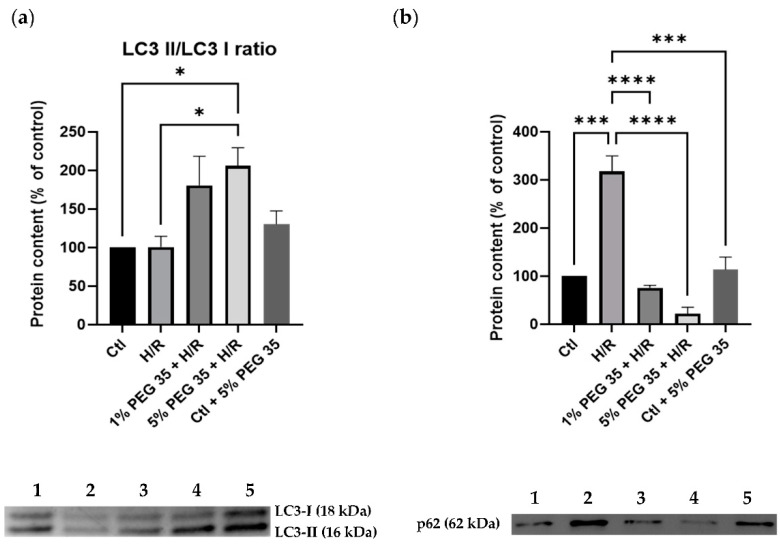
Autophagy-related protein markers in HepG2 cells. (**a**) Representative Western blot for LC3 I and LC3 II and respective LC3II/LC3I ratio quantification. (**b**) Representative Western blot for p62 and respective quantification for: 1-Ctl; 2-H/R; 3–1% PEG35 + H/R; 4–5% PEG35 + H/R; 5-Ctl + 5% PEG35. The values shown represent the mean ± SEM of 3 independent experiments. * *p* < 0.05; *** *p* < 0.001; **** *p* < 0.0001.

Growing evidence has shown the cytoprotective role autophagy plays in maintaining mitochondrial function and cell survival following hepatic ischemia/reperfusion [19]. Therefore, we next investigated whether PEG35 preconditioning induces autophagy following hypoxia/reoxygenation injury. The levels of LC3-II, the active form of LC3, a protein involved in autophagosome formation, exhibited an increase in its content in the presence of 5% PEG35 when compared to the hypoxia/reoxygenation group (Figure 4a). In addition, the analysis of another autophagy marker, p62, showed that cells submitted to hypoxia/reoxygenation have a significant increase in p62 protein content, which was significantly prevented by PEG35 preconditioning (Figure 4b). The augmented LC3II/I ratio and decreased p62 content strongly suggest that, at least in part, PEG35 preconditioning may significantly alleviate hypoxia/reoxygenation injury via autophagy activation.

### 2.5. PEG35 Restored Mitochondrial Biogenesis and Fusion–Fission Dynamics

**Figure 5:**
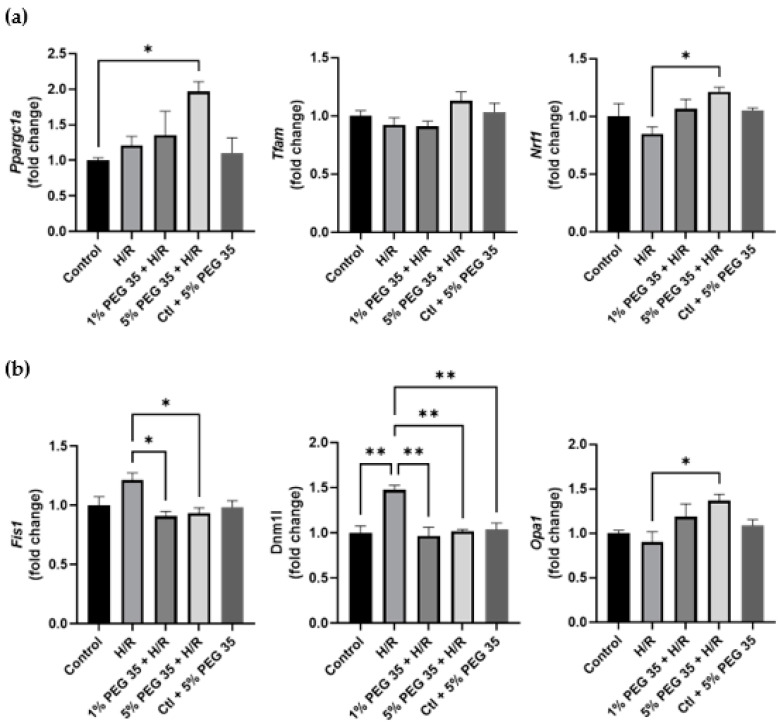
Effect of PEG35 preconditioning on mitochondrial biogenesis and fusion–fission dynamics during HRI. (**a**) Gene expression of proteins involved in mitochondrial biogenesis (*ppargc1a*, *tfam* and *nrf1*). (**b**) Gene expression of mitodynamics-involved proteins, dynamin-related protein 1 (dnm1l), fission protein 1 (*fis1*) and optic atrophy 1 (*opa1*). The values shown represent the mean ± SEM of 3 independent experiments. * *p* < 0.05; ** *p* < 0.01.

Mitochondrial biogenesis is regulated by PGC-1α and the downstream nuclear respiratory factor 1 (NRF1) and transcription factor A (TFAM), controlling mitochondrial turnover, content and number to maintain the metabolic demands. Our study demonstrates a significantly increased expression of both genes coding for PGC-1α and NRF1 (*ppargc1a* and *nrf1*, respectively), upon 5% PEG35 preconditioning (Figure 5a). However, no differences were observed in the *tfam* expression.

Mitochondria are highly dynamic organelles regulated by fission and fusion events. As can be seen in Figure 5b, hypoxia/reoxygenation leads to the upregulation of genes coding for the mitochondrial fission 1 protein (Fis1) and dynamin-related protein 1 (Drp1) (*fis1* and *dnm1l*, respectively), both mitochondrial fission-related genes. Both PEG35 concentrations were able to attenuate this increased gene expression to control levels. In addition, 5% PEG35 preconditioning significantly upregulated the expression of the gene coding for optic atrophy 1 protein (*opa1*), a key mediator in mitochondrial fusion. 

These results suggest that PEG35 preconditioning may protect against hypoxia/reoxygenation-induced mitochondrial dysfunction through the regulation of PGC-1α-mediated mitochondrial biogenesis and the balance of fusion–fission dynamics, resulting in the alleviation of hepatic hypoxia/reoxygenation injury.

## 3. Discussion

Hepatic ischemia/reperfusion injury is the primary cause of liver damage after liver transplantation or hepatectomy. It is characterized by oxidative stress, inflammation and impaired autophagy and mitochondrial function, which leads to hepatocellular damage, contributing to organ failure. During the ischemic period, the deprivation of oxygen leads to the cessation of oxidative phosphorylation, causing ATP depletion, while in the reperfusion period, a burst of mitochondrial ROS production is considered to play a critical role in the organ damage associated with ischemia/reperfusion injury [5].

Several therapeutic strategies have been implemented to counteract the deleterious effects of ischemia/reperfusion injury, including those involving pharmacological conditioning. Polyethylene glycols are linear polymers of ethylene oxide with hydroxyl terminal groups that have been shown to provide beneficial effects by regulating cell survival pathways in different organs [20]. Nevertheless, the exact mechanism of the protective effects of PEG35 on ischemia/reperfusion-induced cellular injury is unclear.

Both in vitro [21] and in vivo [14] studies have demonstrated that PEG preconditioning can protect myocytes from ischemia/reperfusion injury-induced cell death. PEG35 administration has also been described as a protective strategy in other organs, such as the pancreas in an inflammation model [10]. To the best of our knowledge, this is the first study evaluating the effects of PEG35 preconditioning against hypoxia/reoxygenation-induced injury in HepG2 cells. In accordance with the above studies reporting that PEG induces protection from ischemia/reperfusion injury-related cell death, we found that human hepatocytes pretreated with 5% PEG35 and then submitted to hypoxia/reoxygenation presented a higher cell viability compared to cells without PEG35 preconditioning (Figure 1b).

PEG35 has been associated with higher levels of the mitochondrial enzyme ALDH2 and enhanced mitochondrial machinery, culminating in decreased ischemic injury [15,16]. ALDH2 is located in the mitochondrial matrix and is abundantly expressed in numerous organs, including the liver, heart, brain, intestine and kidneys [22,23]. ALDH2 is mainly known for its detoxifying properties, conferring a protective shield against toxic agents, including acetaldehyde (alcohol metabolism), lipid peroxidation-originated products and ROS [15]. In our experimental model, we detected an increased ALDH2 content in the 5% PEG35 group, while in the other groups, the protein levels were similar (Figure 2a).

A pathological increase in ROS production is a hallmark of hepatic ischemia/reperfusion injury, driving hepatocyte death. As expected, the hypoxia/reoxygenation group showed an increase in ROS production, which was significantly reverted in the presence of 5% PEG35 (Figure 2b). This result is in accordance with the increased levels of ALDH2, and its ability to decrease oxidative stress, observed under 5% PEG preconditioning. We also assessed the protein content of Nrf2, which plays an important role in the cellular antioxidant response against multiple stress injury factors. The effective activation of Nrf2 is well known to lead to better outcomes following hypoxia/reoxygenation injury [24]. In the present study, we observed that hypoxia/reoxygenation diminishes the levels of Nrf2 but, despite a tendency to increase Nrf2 content, 5% PEG35 was not able to significantly reverse the hypoxia/reoxygenation effects (Figure 2c). In addition, the gene expression of MnSOD, an essential mitochondrial antioxidant enzyme, was shown to be downregulated upon hypoxia/reoxygenation and upregulated in both PEG35 groups (Figure 2d). PEG35 decreased the generation of ROS during hypoxia/reoxygenation and upregulated the expression of ALDH2 and MnSOD, indicating that PEG35 protected hepatocytes from hypoxia/reoxygenation injury via the upregulation of antioxidant enzymes.

Ischemia/reperfusion is well known to alter the energy metabolism due to the impairment of mitochondrial function [25,26]. The observed excessive ROS production during hypoxia/reoxygenation may lead to the attack of cellular membranes and subcellular organelles, leading to the decrease in mitochondrial membrane potential and consequent mitochondrial dysfunction. In this study we demonstrated that PEG35 preconditioning protected against the loss of mitochondrial function in human hepatocytes during hypoxia/reoxygenation (Figure 3a). Furthermore, the ATP content in HepG2 cells submitted to hypoxia/reoxygenation was found to be decreased (Figure 3b), which is in agreement with the lower mitochondrial membrane potential observed in the same group, suggesting the impairment of mitochondrial function. As expected by the observed recovering of mitochondrial ΔΨ, PEG35 preconditioning efficiently preserved the ATP content following hypoxia/reoxygenation. The results reported here show that PEG35 preconditioning may improve ischemia/reperfusion injury by preserving mitochondrial function and decreasing excessive ROS production and ATP depletion, as well as recovering the membrane potential. 

Recently, Bardallo et al. [27] reported better liver protection against ischemic insult through the reduction of oxidative stress via ALDH2 upregulation and the enhancement of cytoprotective autophagy in a PEG35-dependent manner. Autophagy is a crucial process in the clearance of dysfunctional organelles, and it has been generally recognized as a protective process in response to various intra- and extracellular stimuli, including ischemia/reperfusion injury [28]. Enhancing autophagy ameliorates hepatic function by eliminating the dysfunctional mitochondria [4], while autophagy inhibition increases mitochondrial oxidative stress and triggers cell death during liver ischemia/reperfusion injury [29]. In accordance with this, Wang and colleagues reported that increasing autophagy alleviates hepatic injury and improves mitochondrial function against ischemia/reperfusion injury [30]. LC3 is a key player in the autophagic processes, which also includes mitophagy. During autophagic events, the soluble form (LC3-I) is converted into LC3-II, which is recruited to the autophagosome formation [31]. Furthermore, p62, also known as sequestosome 1, is another key protein involved in the autophagic process that targets specific cargoes for autophagy. Under normal conditions, basal autophagy clears p62 and its associated cargo. On the other hand, under conditions of decreased/deficient autophagy, p62 and its associated cargo accumulate in the cytoplasm [32]. In Figure 4a, we observed that PEG35 preconditioning increased the conversion of LC3-I into LC3-II. In agreement with this, HepG2 cells submitted to hypoxia/reoxygenation showed an increased accumulation of p62, while HepG2 cells exposed to PEG35 prior to hypoxia/reoxygenation presented a strong decrease in p62 content (Figure 4b). The increased levels of LC3 II and p62 degradation suggest an autophagy enhancement followed PEG35 preconditioning, which may contribute to the elimination of dysfunctional mitochondria during ischemia/reperfusion injury, leading to improved mitochondrial performance.

PGC-1α is a master regulator of mitochondrial biogenesis that enhances different transcription factors, such as NRF1 and TFAM, which control mitochondrial turnover, content and number to maintain the metabolic demands [4]. Previous studies reported enhanced mitochondrial functioning following hepatic ischemia/reperfusion injury through the stimulation of mitochondrial biogenesis via the induction of the PGC-1α/NRF1/TFAM pathway [4,33,34]. In accordance, in our study, 5% PEG preconditioning upregulated the expression of *ppargc1a* and *nrf1* (Figure 5a), indicating an enhancement of mitochondrial biogenesis. 

Mitochondria are dynamic organelles, continuously dividing and elongating through frequent fusion and fission in response to cellular stress and consequent alterations in the intracellular environment [35]. Mitochondrial fusion and fission are two essential quality control mechanisms which favor the segregation and clearance of dysfunctional mitochondria to achieve homeostasis. Compelling evidence of the interplay between mitochondrial quality control and cell fate in different organs subject to ischemia/reperfusion has been gathered over the last few years [8]. Modifications to the regulators involved in fission or fusion processes, the loss of cristae integrity, alongside the inefficient removal of damaged mitochondria, have all been implicated to play a vital role in ischemia/reperfusion injury [36]. OPA1 is a crucial protein involved in mitochondrial fusion which may interact with various numbers of mitophagy receptor proteins in order to collaborate in mitochondrial dynamics and mitophagy [37,38]. OPA1 knockdown has been reported to exacerbate the deleterious effects provoked by ischemia/reperfusion [39,40]. Moreover, increased mitochondrial fusion and mitophagy through the AMPK-OPA1 signaling pathway was observed to protect against cardiac ischemia/reperfusion injury, whereas OPA1 knockout abolished the protective effects [41]. In here, we report an increased expression of *opa1* after 5% PEG35 preconditioning (Figure 5b). It has been demonstrated that the loss of mitochondrial membrane potential triggers OPA1 proteolysis and inhibits mitochondrial fusion [42,43]. As mentioned above, our results demonstrate that 5% PEG35 preconditioning increased mitochondrial membrane potential when compared to hypoxia/reoxygenation, which is in accordance with the effects of increased *opa1* upregulation. 

Contrary to fusion, mitochondrial fission has been reported to be triggered by ischemia/reperfusion injury [44,45]. Mounting evidence confirms that the inhibition of mitochondrial fission might protect several tissues from ischemia/reperfusion injury [46]. Excessive mitochondrial fission has been related to mitochondrial fragmentation and the triggering of cell apoptosis [47]. Drp1 and Fis1 are essential proteins involved in the fission processes [36,48]. Bi et al. [49] have reported increased levels of Drp1 and Fis1 after hepatic ischemia/reperfusion. 

Nevertheless, pharmacological postconditioning efficiently decreased the levels of these two mitochondrial fission-related proteins. Furthermore, the inhibition of Drp1 led to increased mitophagy and the consequent clearance of damaged mitochondria and the prevention of ROS production in cardiac ischemia/reperfusion injury [50]. In our study, cells submitted to hypoxia/reoxygenation significantly increased the expression levels of *dnm1l* and *fis1*, and this upregulation was attenuated by PEG35 preconditioning (Figure 5b). We understand that it is reasonable to assume that changes in mRNA expression will have corresponding changes in protein levels. However, the correlation between proteins and their mRNA levels are sometimes poor [51,52].

Taken together, our findings suggest that PEG35 preconditioning regulates hypoxia/reoxygenation injury-induced imbalances in mitochondria dynamics by elevating mitochondrial fusion and diminishing mitochondrial fission.

**Figure 6:**
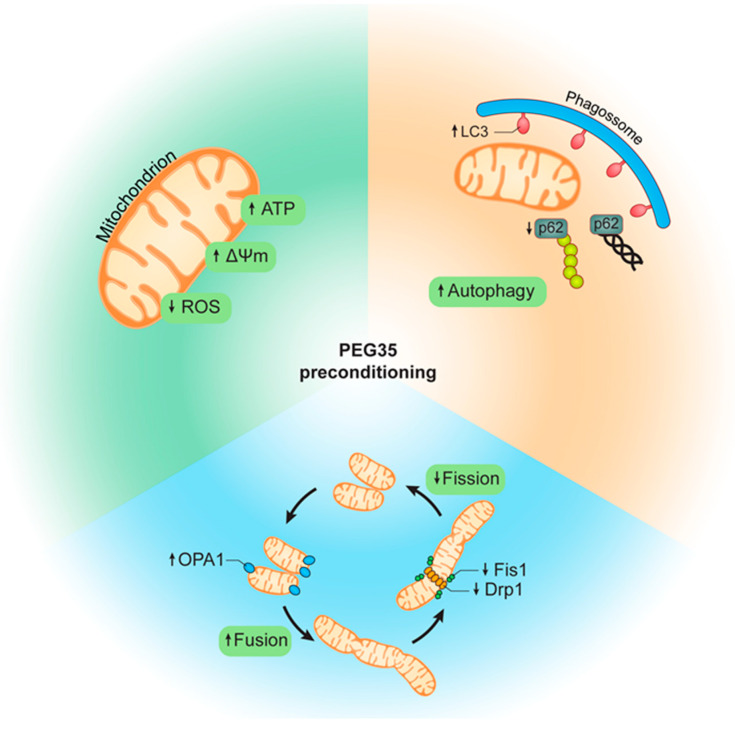
Schematic representation of PEG35-mediated protection following hypoxia/reoxygenation injury. PEG35 preconditioning alleviates mitochondrial dysfunction, including the recovery of mitochondrial membrane potential and ATP levels and the reduction of ROS production. In addition, PEG35 treatment suggests enhanced autophagy and a modulation of mitochondrial biogenesis via increased mitochondrial fusion and decreased mitochondrial fission following hypoxia/reoxygenation injury.

In summary, in the present study we used an in vitro model to explore the possible protective molecular mechanisms of PEG35 preconditioning on diminishing hypoxia/reoxygenation injury. We observed that 5% PEG35 preconditioning presented a better outcome in most of the parameters analyzed when compared to 1% PEG35 preconditioning, showing that PEG35 protective effects are dose dependent. We demonstrated that 5% PEG35 preconditioning efficiently attenuates hepatic hypoxia/reoxygenation injury by alleviating mitochondrial dysfunction via the enhancement of autophagy and mitochondrial biogenesis and dynamics (Figure 6). 

In conclusion, PEG35 preconditioning seems to be a viable strategy to confer hepatocellular protection against ischemia/reperfusion injury and, therefore, we suggest that PEG35 might be considered to be a suitable pharmacological preconditioning agent in liver surgery.

## 4. Materials and Methods

### 4.1. Cell Culture

HepG2 cells were cultured in 75 cm^2^ flasks (Sarstedt, Nümbrecht, Germany) with 15 mL Dulbecco’s modified Eagle medium (DMEM, Sigma-Aldrich, St. Louis, MO, USA), supplemented with 1% antibiotic–antimycotic (penicillin/streptomycin/amphotericin B; Gibco, Waltham, MA, USA) and 10% fetal bovine serum (FBS; Invitrogen, Waltham, MA, USA) in a humidified 5% CO_2_ atmosphere at 37 °C. When cells reached 70–90% confluence, they were detached with TrypLE Express (Gibco, Waltham, MA, USA) and subsequently counted using the trypan blue dye exclusion technique and plated in 12-well plates.

### 4.2. Cell Culture

HepG2 cells were placed in a hypoxia-mimetic solution (137 mM NaCl, 12 mM KCl, 0.9 mM CaCl2, 0.49 mM MgCl2, 4 mM HEPES, 20 mM lactate, 10 mM deoxyglucose, 0.75 mM sodium dithionite, pH 6.5) to induce hypoxia [53,54]. To attempt to simulate ischemic conditions, we used a hypoxia medium because it is a solution free of metabolic substrates. It was supplemented with lactate to simulate its accumulation due to anaerobic glycolysis, and deoxyglucose to inhibit glycolysis and further shut down cellular metabolism [55]. In addition, sodium dithionite is a powerful oxygen scavenger, and consequently leads to rapid oxygen depletion from the solution, as well as a rapid reversibility of its effects during the washout [56]. Following the hypoxia time, reoxygenation was induced via hypoxia medium exchange to complete the growth medium (DMEM).

**Figure 7:**
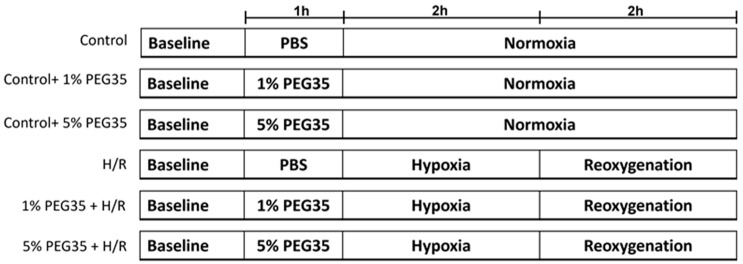
A schematic representation of the experimental protocol. HepG2 cells were randomly divided into the following groups: control, control treated with either 1% PEG35 (Ctl + 1% PEG35) or 5% PEG35 (Ctl + 5% PEG35), 2 h of hypoxia followed by 2 h of reoxygenation (H/R) and preconditioning with 1% PEG35 or 5% PEG35 prior to H/R, 1% PEG35 + H/R and 5% PEG35 + H/R, respectively.

For PEG35 preconditioning, cells were pretreated with PEG35 diluted in PBS for 1 h prior to the induction of hypoxia, at 2 different concentrations, 1% or 5% PEG35 (Figure 7).

### 4.3. MTT Assay

Cellular viability was determined through the evaluation of cellular reductive capacity with the reduction of MTT (3-(4,5-dimethylthiazol-2-yl)-2,5-diphenyltetrazolium bromide) to its insoluble formazan crystals, as previously described [57]. HepG2 cells were seeded in 12-well plates and allowed to attach for 1 day prior the assay. After the corresponding treatments, MTT solution (5 mg/mL in PBS) was added to the cells for 3 h. Afterwards, the incubation media were discarded, and formazan crystals were dissolved in isopropanol. A crystal-dissolved solution of each sample was quantified via spectrometry (540 nm) using a Victor plate reader (Perkin-Elmer, Waltham, MA, USA).

### 4.4. Measurement of Mitochondrial Membrane Potential (ΔΨm)

HepG2 cells was measured with a fluorescent probe, tetramethylrhodamine methyl ester (TMRM), as described before [57]. Briefly, after the hypoxia reoxygenation protocol, cells were incubated with 6.6 µM TMRM for 15 min at 37 °C. Afterwards, cells were washed, and culture medium without FBS and phenol red was added. Fluorescence was assessed using the excitation and emission wavelengths of 485 and 590 nm, respectively.

### 4.5. Measurement of ROS Production

H2DCFDA oxidation to 2,7-dichlorofluorescein (DCF) by ROS was measured as an indicator of ROS accumulation, as before [58]. Briefly, cells were incubated under the same conditions described in the previous section. Then, cells were loaded with 50 µM H2DCFDA for 30 min at 37 °C, and washed and placed in a culture medium without FBS and phenol red. The fluorescence resulting from the formation of oxidized derivatives was monitored at an excitation wavelength 485 nm and an emission wavelength 538 nm for 10 min to calculate the rate of ROS formation.

### 4.6. ATP Content

HepG2 cells were cultured in 6-well plate under the same conditions described in the experimental protocol in Section 4.2. At the end of the treatment, the extraction of ATP from cells was performed as previously described [59]. Briefly, cells were washed and then scraped in PBS 1X at 37 °C. Cells were centrifuged at 1000× *g* for 3 min and the pellets were resuspended in 25 µL of KOH buffer (KOH 2.5 M, K2HPO4 1.5 M) and 75 µL of H_2_O. After sonication and centrifugation at 18,000× *g* at 4 °C for 2 min, the pH of the supernatant was adjusted to 7 with KH_2_PO_4_ 1 M, and the pellet was stored at −20 °C for protein quantification. 

An ATP Bioluminescent Assay Kit (Sigma-Aldrich, St. Louis, MO, USA) was used to measure the ATP content in each sample, according to the supplier’s instructions. Bioluminescence was measured using a Victor3 plate reader (PerkinElmer, Waltham, MA, USA).

### 4.7. Quantitative Real-Time PCR

Total RNA was extracted from HepG2 cells using the PureLink RNA Mini Kit (Invitrogen, Waltham, MA, USA) according to the manufacturer’s recommendations. RNA was quantified with a Nanodrop One (Thermo-Fisher, Waltham, MA, USA) and 10 ng of RNA was reverse transcribed using the iScript cDNA Synthesis kit (Bio-Rad, Hercules, CA, USA), according to manufacturer’s instructions. Then, cDNA was diluted 1:10 and SsoAdvanced Universal SYBR Green Supermix (Bio-Rad) was used for qPCR reactions. 

The expression level of target genes was calculated using the 2^−ΔΔCt^ transformation method [60] and normalized to the housekeeping gene 18S rRNA. The primers used are shown in the Table 1.

### 4.8. Western Blot Analysis

After treatments, cells were washed twice and scraped in 1 mL of ice-cold PBS 1X. Cells were centrifuged for 3 min at 10,000× *g* at 4 °C and the pellets were resuspended in an ice-cold RIPA lysis buffer supplemented with protease inhibitors (Thermo Scientific). Lysates were sonicated and centrifuged for 10 min at 12,000× *g* at 4 °C. The protein concentration was quantified via the bicinchoninic acid assay and subsequently mixed with Laemmli buffer containing 8% β-mercaptoethanol and denatured at 80 °C for 5 min. Then, 50 µg of protein was separated using TGX Stain-Free polyacrylamide gels (Biorad), according to the manufacturer’s recommendations. Gels were activated using a GelDoc EZ (Bio-Rad) and the proteins were subsequently transferred to a nitrocellulose membrane using a Trans-Blot Turbo Transfer System (Bio-Rad). Membranes were blocked for 2 h in 5% non-fat dry milk and incubated with the specific primary antibody at 4 °C overnight. Membranes were washed with TBS-T and incubated for 1 h with secondary antibodies. After washing the membranes with TBS-T, they were revealed using a ChemiDoc MP (BioRad). Total protein quantification of the respective lanes was used to perform blots normalization, following standard procedures [61,62]. Images were analyzed using Image Lab 6.1 Software (Bio-Rad). The antibodies used are listed in Table 2.

### 4.9. Statistical Analysis

Data are presented as mean ± S.E.M. A one-way ANOVA with Tukey’s test was performed for the evaluation of statistical significance (*p* < 0.05). Statistical analysis was performed using GraphPad Prism 9.0.0 (GraphPad Software, San Diego, CA, USA).

## Figures and Tables

**Table 1:** Nucleotide sequences of primers used in qPCR.

Gene	Sequence	NCBI’s Nucleotide Accession Number
*Dnm1l*	AAG AAC CAA CCA CAG GCA AC GTT CAC GGC ATG ACC TTT TT	NM_012062.4
*Fis1*	TTA TTT ACA CTC ATC CCA AAG C CTG TCC TTT CCC TGT TCT C	NM_016068.2
*Mnsod*	GGA AGC CAT CAA ACG TGA CT CTG ATT TGG ACA AGC AGC AA	NM_000636.2
*Nrf1*	GAA TTG CCA ACC ACG GTC AC GCG CCA TAG TGA CTG TAG CT	NM_005011.4
*Opa1*	CAG AAA GAT GAC AAA GGC ATT C GCA ATC ATT TCC AAC ACA CTA G	NM_015560.2
*Ppargc1a*	CCT TGC AGC ACA AGA AAA CA CTG CTT CGT CGT CAA AAA CA	NM_013261.3
*Tfam*	CCG AGG TGG TTT TCA TCT GT ACG CTG GGC AAT TCT TCT AA	NM_003201.2
18S rRNA	AAC GGC TAC CAC ATC CAA TTT TCG TCA CTA CCT CCC	NR_003286.2

**Table 2:** Primary and secondary antibodies used in Western blot analysis.

Antibody	*M_W_* (kDa)	Dilution	Supplier	Reference Number
		*Primary antibodies*		
ALDH2	56	1:1000	Abcam	ab194587
LC3	18, 16	1:1000	Sigma-Aldrich	L7543
Nrf2	75	1:1000	Millipore	ABE413
p62	62	1:100	Santa Cruz	sc-84618
		*Secondary antibodies*		
StarBright Blue 520, Goat Anti-Rabbit IgG	-	1:5000	Bio-Rad	12005870
StarBright Blue 700 Goat Anti-Rabbit IgG	-	1:5000	Bio-Rad	12004162

## Data Availability

All data are available upon reasonable request to the corresponding author.
